# Application and optimization of an artificial neural network to forecast thermo-mechanical behaviour of HLE steel under weld spot effect

**DOI:** 10.1016/j.heliyon.2023.e16739

**Published:** 2023-06-05

**Authors:** Brahim Belahcene

**Affiliations:** Abou Bekr Belkaid University, Tlemcen, Algeria

**Keywords:** Thermo-mechanical, Finite element method, ANN model, Numerical twin

## Abstract

The reliability of assembled structures is significantly influenced by the applied thermomechanical stresses and the robustness degree of the simulation numerical methods. The utilization of classical numerical methods such as the finite element method (FEM), extended finite element method XFEM, and mean weighted residuals method are computational costs due to the complexity of the materials behaviour laws, physicals mathematical model and laboratory apparatus cost. To ensure accurate investigation techniques, it should be performed a numerical model used for resolving welding physical equations governed. The main objective of this study is to architect and optimize an intelligent model based on an artificial neural network to resolve a complex model of the calculation effect of spot welding on the behaviour of HLE steel. The ANN model gives a strong correlation between the dataset as numeric input and the target. The artificial neuron network gives a proxy model approach to exploit input data and results extracted by simulation of weld spot using finite element method FEM. The performance evaluation of the ANN model was carried out using mean square error and regression analysis. As a result, the present model ANN gives with minimum computational cost a good match of temperature estimating, equivalent stress and strain along the contact area of two thin plates of steel studied assembled by weld spot with a comparison between classical models using FEM.

## Highlights on proposed approach

➢ Fast tool for assessment of materials behaviour
➢ Minimum competition time
➢ Give forward and backward investigation on the physical model by estimation or prediction parameters
➢ Forecast behaviour of materials
➢ Carry out the solution of complex problems with minimum computational cost.
➢ Give fast decisions to make predictive control and monitor of material behaviour with the variation of the operators parameters.

## 1 Introduction

The study of assembly techniques has been widely used for designing high-resistance materials to perform their properties that are used in industrial sectors such as automotive manufacturing, the oil and gas industry … etc. The behaviour of assembly metal structures is governed by conditions and parameters of the welding process. Some interrelationships between fusion zone (FZ), electric conductance and contact zone radius are examined [1]. The uniaxial tensile testing and temperatures of stainless steel are correlated [2]. The welding resistance affects microstructure gradient and metallurgical transformations [3]. The heat distribution during welding spots can be altered by the rapid movement of the molten metal [4]. Furthermore, the anode speed movement has a significant effect on assembled metals [5]. Therefore, the cooling system by using water during the welding process is mandatory because it affects the distribution of stress and the thermal field [6]. Herein, the multi-coupled model procedure for spot welding is described in Refs. [7,8]. The thermal transient can affect the electrodes of the welding spot [9]. The failure modes of the dissimilar thickness dual-phase sheets are investigated in Ref. [10]. The real-time monitoring of the spot-welding process is discussed in [11,12]. Moreover, the chemical compositions can have significant effects on the microstructure and properties of HAZ [13]. The mechanical properties and microstructures for acicular ferrite steel under controlled thermomechanical process parameters are reported in [14]. A correlation between the corrosion damage behaviour and thermal treatment of HLE steel is reported in [[15], [16], [17]].

These previous studies cited concerning assemblage techniques, spot welding modelling and simulation are based on conventional complex models and methods using numerical methods (FEM) as simulation and several apparatuses. Furthermore, the analytical models have based on restricted parameters, and sometimes it isn't easy to apply them in other fields. Because they base on empirical constants and laboratory data set. However, it should be insert new techniques to carry out the solution of complex problems with minimum computational cost.

The artificial neural network “ANN” architecture technique contains many nodes, connections, and hidden layers [18]. The basic principle of ANN is based on input data or signal (neuron input), weight function and output results (neuron output) [18].

The use of an artificial neural network (ANN) based flow law for modelling thermomechanical solicitations is reported by Pantalé, O [20]. However, the author used in the training part data established by Ji et al. [21] and multi-laye multi-layer feed-forward for model architecture reported by Hornik et al. [22]. Wherein, the things that distinguish present research are evident in the privacy of the dataset or input data used in the trained model which carried out in our previous studies (B. Belahcene) [[15], [16], [17]] also the present hybrid model FEM&ANN.

This literature review is deeply analyzed by exploring references networks connectivity (Appendix A). Thus, it improved by many references related with aim of the research subject which allows us to formulate the primary problem statement and to open the gate for the projection of these ideas in this research field as compiling the numerical twin or hybrid numerical model proposed.

In the present research, hybrid ANN has been used for forecasting the behaviour of HLE Steel under the weld Spot effect. The ANN models’ performance has been evaluated with numerical data generated by FEM. To optimize ANN, diverse effective factors have been tested. The performance of the hybrid model was evaluated through the coefficient of determination (R2), mean squared error (MSE), and mean squared error (MAE), Jacobian calculations (Levenberg-Marquardt) … as reported in the present procedure used for the application and optimization of the ANN model (Fig. 3).

Thus, the novelty of this work is to make a numerical twin model that minimizes computational cost and forecast distribution of temperature, stress and strain equivalent along nugget (FZ) and affected zone (HAZ). The hybrid model FEM&ANN can be unconventional for predicting material behaviour under applied loads. Likewise, it can give fast decisions to make predictive control and monitor material behaviour with the variation of operator parameters.

## 2 Computational modelling

### 2.1 Numerical modelling by finite elements

**Fig. 1:**
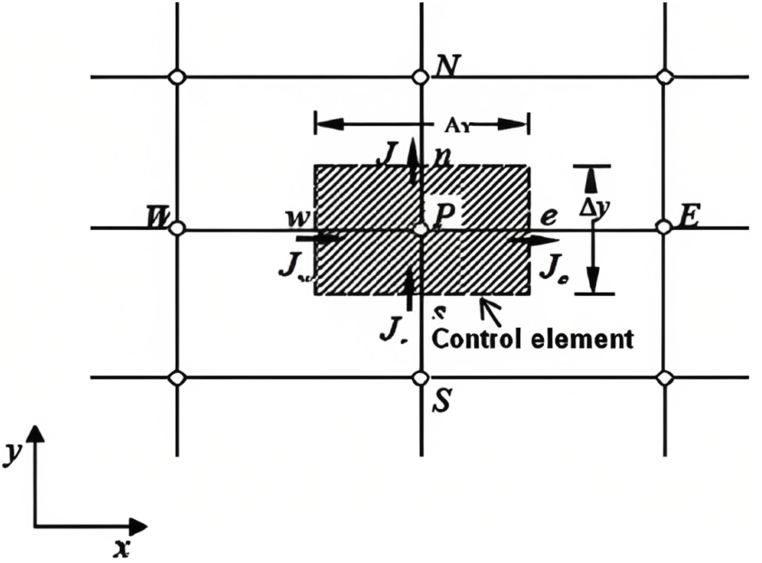
Integrate solution by control element.

A physical system can be modelled with one-order differential equations depending on its degree of freedom and its dimensions. One of the digital methods of resolution is the finite element method. It allows for resolving the governing differential equations or the strong form to the weak form before achieving physical solutions. The structure is discretized into form geometric elements whose nodes are the assembly points of other neighbours elements. The constraints applied directly to these nodes lead to solutions in the mesh elements. The fineness of the mesh refines the solutions. The finite element method includes two formulations; one is based on the direct variational method like that of Rayleigh-Ritz and the other on the method of mass residues like that of Galerkin. The stationarity condition leads to the derivative of the fundamental equations of the variational method for problems at limit values. It has the advantage of subtraction of derivative functions [23]. This method uses mesh elements as well as linear, triangular, and quadratic shapes to present the variables to be searched. The numerical solution is transformed into a physical variable. The differential equation presented is transformed into the weak form after a certain approximation made by partial derivatives. The integrated solution can be checked by the control element as presented in Figure (1). The final solution sometimes does not match the measured solution that has to the residue or remains of the derivative. The residual is contracted to multiply by functions of weights. The discretization must be ensured under the following conditions: Existence of solutions, uniqueness of the solution, stability, convergence, and error estimation of the discrete solution.

The physical problem equations can be written in the following form

(1.1) $$\frac{\partial }{\partial \left(x_{ij}\right)}\left(F\right)=\frac{\partial }{\partial \left(x_{ij}\right)}\left(\frac{\partial \left(F\right)}{\partial \left(x_{ij}\right)}\right)+S\varphi$$

Consider the control element in Fig. 1.

The integration of the equation on the control element given in (1.2):

(1.2) $$J_{e}-J_{w}+J_{n}-J_{s}=\int_{V}S_{\varphi }\mathrm{d}V$$

where the variables J e, J w, J n and J s are the control elements of the integrated solution on the faces in three directions e, w, n,s. The weak form is described in matrix form through:

(1.3) {F}=[k]⋅{δ}

A numerical model (finite element method FEM) is used for the first time based on the asymmetry model. Therefore, the boundary conditions limits are applied and an assessment of mechanical behaviour under the used loads is carried out before being used in the ANN model as the target to make a digital win simulation.

### 2.2 Governing equations

The spot welding process can be simulated as an axisymmetric problem. The transient thermal governing equations (1.4), (1.5) is assumed by axisymmetric.

(1.4) $$q=-k\frac{\partial T}{\partial n}$$

(1.5) $$\frac{\partial }{\partial r}\left(C_{e}\;\frac{\partial \emptyset }{\partial r}\right)+\frac{C_{e}}{r}\frac{\partial \emptyset }{\partial r}+\frac{\partial }{\partial z}\left(C_{e}\;\frac{\partial \emptyset }{\partial z}\right)=0$$

The thermoelectric model is governed by the following matrix equation (1.6):

(1.6) $$\left[C^{t}\right]\;\left\{\dot{T}\right\}+\left(\left[K^{t}\right]\;\left\{T\right\}+\left[K^{v}\right]\left\{V\right\}\right)=\left\{\begin{array}{c} \left\{Q\right\}\\ \left\{I\right\} \end{array}\right\}$$

The equilibrium equation is given by the next equation (1.7) to make a structural analysis.

(1.7) ∇σ(r,t)+b(r,t)=0

(1.8) $$d\left\{\sigma \right\}=\left[D\right]d\left\{\varepsilon \right\}+\left[D^{e}\right]\left(\left\{\alpha \right\}+\frac{\partial {\left[D^{e}\right]}^{-1}}{\partial T}\left\{\sigma \right\}\right)dT$$

The thermo-elastic-plastic model is given by equation (1.8)

### 2.3 Geometric model

**Fig. 2:**
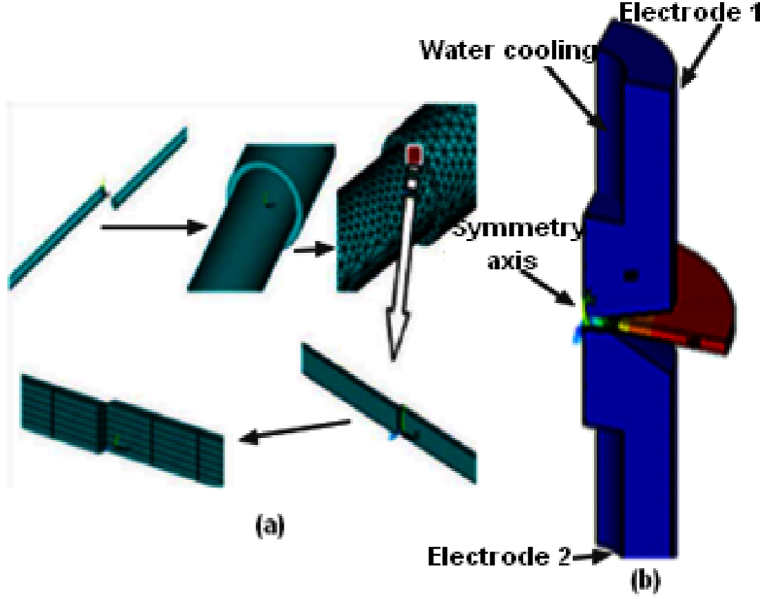
(a) Upscaling structure; (b) Asymmetry model.

Two rectangular steel plates are designed with the following dimensions: half-height (Hp 1/2 = 0.75 mm); half-length (L1/2 = Wp 1/2 = 7.5 mm). The plates are loaded by using two copper electrodes that have the same contact rayon dimension (3 mm); they are cooled by water liquid. One quarter (1/4) of the structure was presented in Fig. 2, for the reason of symmetry in conditions limits and geometry.

**Fig. 3:**
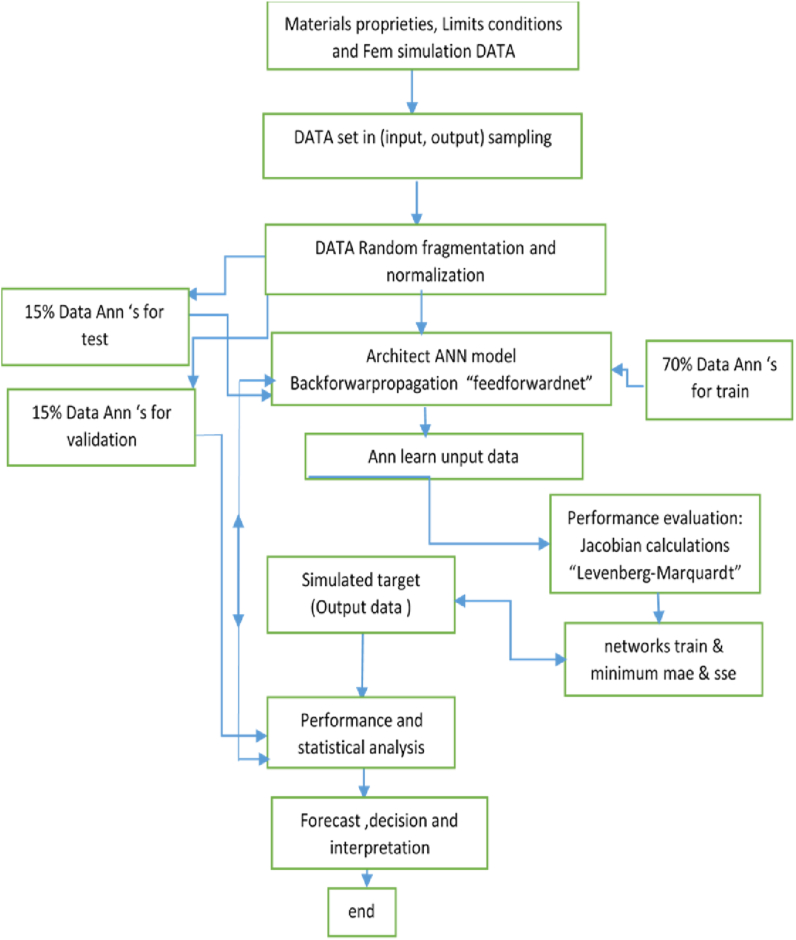
Procedure used for the application and optimization of the ANN model.

### 2.4 Boundary conditions limit

The geometric model of the resistance welding process is shown in Fig. 2; it has a symmetry axis. The boundary conditions are given in Table 1.

**Table 1:** Conditions limit.

Boundary	Mechanical	Thermal	Electrical
Water cooled		18°-25 °C	
Symmetry axis	U = 0	Adiabatic	I = 0
Electrode 1	0.3–0.35 KN	18°-25 °C	V (1.5–2.5)
Electrode 2	U = 0	18°-25 °C	V = 0

### 2.5 Hybrid model FEM&ANN

#### 2.5.1 Procedure of calculation

The calculation technique is shown in Fig. 3, it is based on preparing and sampling data before making a reliable database. Likewise, elaboration and optimization of the ANN model can forecast material behaviour under operator conditions harmonized with the simulation model. Then, by making a comparison between studies get by FEM and target values “output” estimated by ANN. Moreover, a statistical analysis is applied to evaluate the performance of the model and algorithm selected in the simulation.

#### 2.5.2 Network architecture

**Fig. 4:**
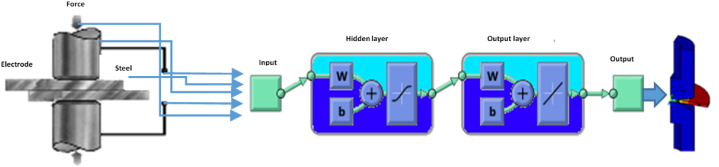
Workflow numerical twin.

The present model is trained using a Feedforward neural network where some layers are optimized with sigmoid and linear output neurons. Ten (10) hidden layers are set in fitting neuro network Fig. 4. To perform the algorithm, many scenarios are made to optimize some hidden layers that can give a good performance of the present model.

In this study, the FEM&ANN settings and methods include adjusting the parameters of the hybrid model to optimize the performance of results. The data preprocessing and feature selection techniques are used for improving the performance of the hybrid model FEM&ANN.

Our technique comprises adjusting the number of layers and hidden layers, the size of each layer, and the type of activation functions used. Moreover, my strategy is to adjust the number of layers, neurons, and activation functions, as well as the learning rate.

The training method of ANN is based on function fitting to make a correlation between the data set of inputs and target outputs.

The data set input is divided into four samples, where 70% of data are set for training algorithm and 15% of data for validation then 15% for testing.

## 3 Results and discussion

**Fig. 5:**
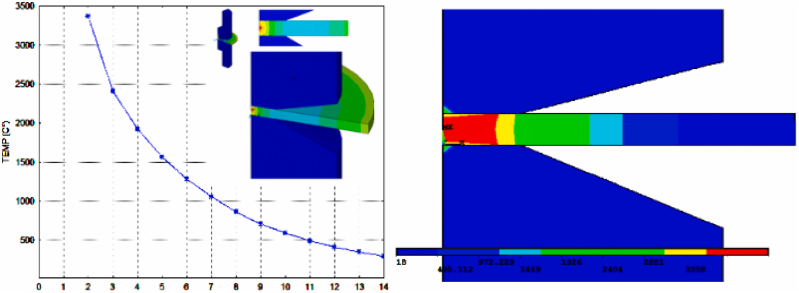
Temperature distribution using FEM.

Thermomechanical behaviour results of thin assembly steel layers are carried out using a hybrid model in comparison with FEM to reach the goal of the analysis. Firstly, the effect of resistance to electric current passage carried out using FEM is illustrated in Fig. 5.

**Fig. 6:**
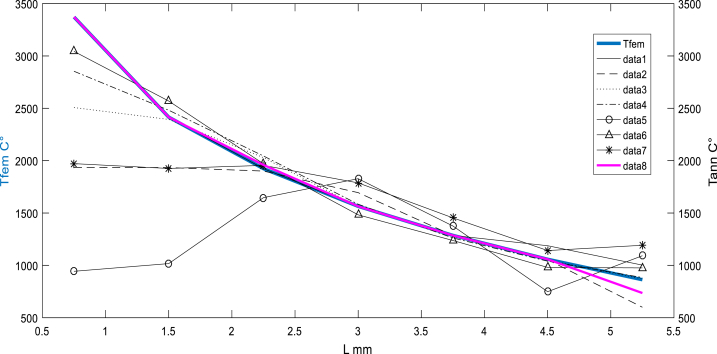
Before sampling: Temperature distribution “target” (simulated by FEM) vs multi scenarios of Temperature distribution using ANN.

**Fig. 7:**
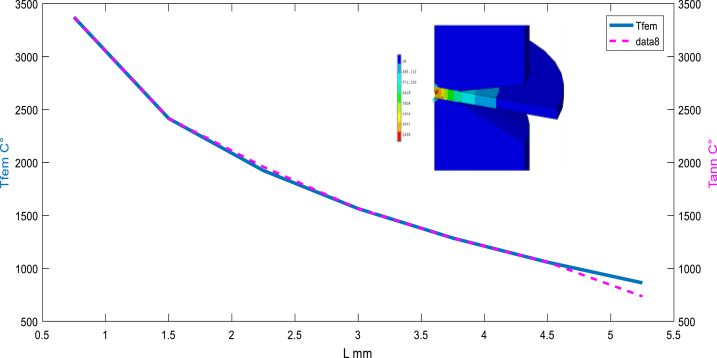
Before sampling: Temperature distribution (simulated by FEM) vs multi scenarios Temperature distribution using ANN (feedforward at the layer N°8).

Fig. 5, Fig. 6, Fig. 7 present the variation of temperature at the assembly area along the nugget zone and affected zones. The average temperature estimated for the nugget zone is about 3367C° (liquid phase). It can be noticed that the nugget (FZ) can't be formed below the melting temperature of 2400C° for the studied material.

**Fig. 8:**
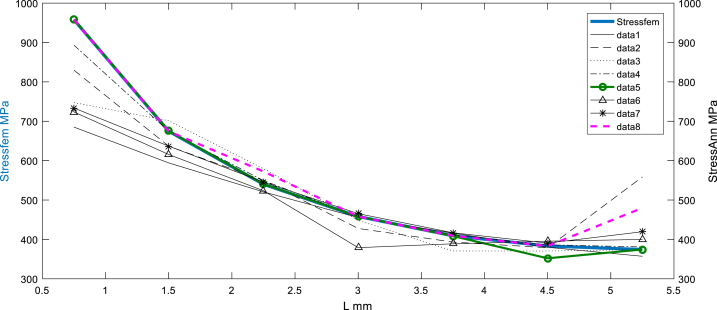
Before sampling: Stress equivalent (simulated by FEM) vs multi scenarios Stress equivalent using ANN.

**Fig. 9:**
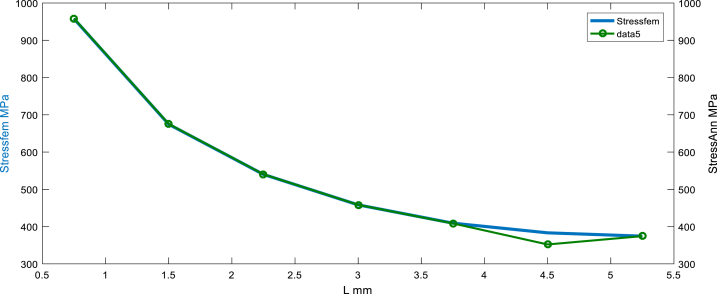
After sampling: Stress equivalent (simulated by FEM) vs Stress equivalent using ANN (feedforward matched at the layer N°5).

Fig. 8, Fig. 9 show the variation of equivalent stress along thin assembly contact at the horizon chosen, wherein, the compressive stress in the assembly area is very important and the average equivalent stress at FZ is about 960 MPa; this is due to the internal generation of thermal and residual stress.

**Fig. 10:**
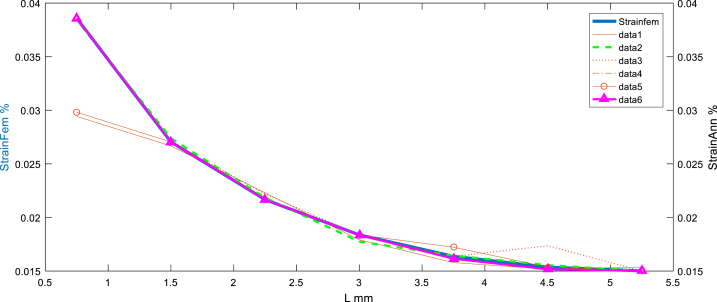
Before sampling: Strain equivalent target (simulated by FEM) vs Stress equivalent using ANN.

**Fig. 11:**
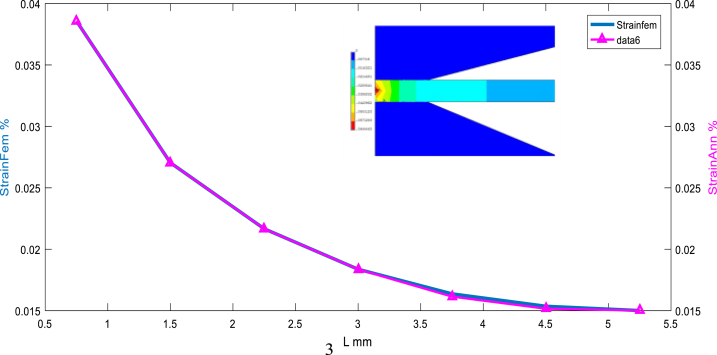
After sampling: Strain equivalent (simulated by FEM) vs Stress equivalent using ANN (feedforward matched at the layer N°6).

The distribution and change of the equivalent strain at the horizon chosen are very significant, and the maximum strain is about 3.7% as it is illustrated in Fig. 10, Fig. 11.

## 4 Conclusions

In this study, we presented the modelling of spot weld by simulation using FEM and hybrid model ANN&FEM. A two-dimensional asymmetric model was used to analyze the effect of resistance weld parameters on the thermomechanical behaviour of an HLE steel. It was shown that the weld parameters have an influence on thermomechanical behaviour on FZ, HAZ and base metal, they have also an influence on the ability of a material to resist variation of internal stress. Moreover, the hybrid technique presented can be used to estimate optimal welding parameters for making an efficient welding control process and reducing cost operations. Correspondingly, the numerical twin presented by the hybrid model FEM & ANN gives good promoted results that allow team engineers to minimize computational cost and forecast the behaviour of materials. Likewise, it can give fast decisions to make predictive control and monitor materials behaviour with the variation of operator's parameters, as well it can give forward and backward investigation on the physical model by estimation or prediction parameters. As a suggestion, in this present study, we look after establishing continuous research on the impact of the selective medium on the behaviour HLE steels on the robustness of the hybrid model ANN&FEM. The corrosion phenomena can be inserted in the dataset as numeric input for enhanced output results and increase strength of the forecast model. In addition, the experimental investigation of the effect of the selective medium on HLE steels have are reported in our last study [15,16,24,19].

## Author contribution statement

BRAHIM BELAHCENE: Conceived and designed the experiments; Performed the experiments; Analyzed and interpreted the data; Contributed reagents, materials, analysis tools or data; Wrote the paper.

## Data availability statement

Data will be made available on request.

## Additional information

Correspondence and requests for information should be addressed to B.B.

## Declaration of competing interest

The author declares that they have no known competing financial interests or personal relationships that could have appeared to influence the work reported in this paper.

Elsevier is the current employer of Dr. Madhuprasad. Dr. Madhuprasad confirms that the research was completed before this author's employment at Elsevier and that the peer review was fully independent of this person.

## Figures and Tables

**Figure:**
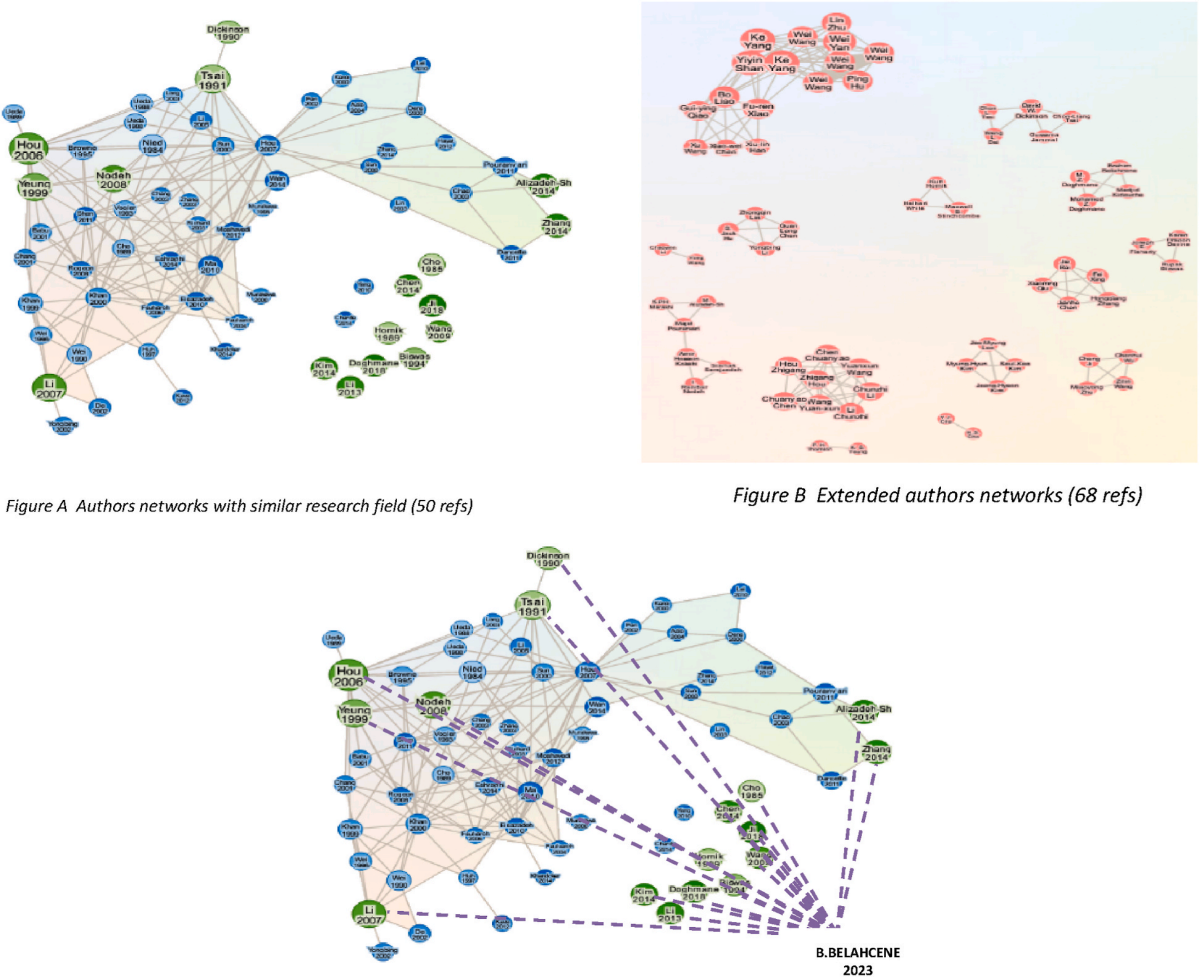


## Nomenclatures

HLE: High limit elasticity
FEM: Finite element method
FZ: Nugget zone
HAZ: Heat affected zone
BM: Base metal
Lp: Lenght of simple (mm)
Wp: Wide of sample (mm)
T: Temperature (C°)
σ: Equivalent stress (MPa)
ε: Equivalent strain
ρ: Density
C: Specific heat capacity
{I}: Current vector
q: Heat flow
{V}: Electric potential vector
Ce: Electrical conductivity
φ: Electric potential
[Ct]: Heat capacity matrix
[Kt]: Thermal conductivity matrix
[Kv]: Electric coefficient matrix
{T}: Temperature vector
b: Load (N)
{σ}: Stress vector
{Q}: Heat flux vector
[D]: Elasto-plastic matrix
{ε}: Strain vector
[De]: Elastic matrix
{α}: Thermal coefficient expansion
{δ}: Vector of displacements
[K]: Stiffness matrix (constant)
{F}: Matrix of the nodal forces (vector of the loadings)
